# *Mycobacterium gordonae*-induced humidifier lung

**DOI:** 10.1186/s12890-015-0107-y

**Published:** 2015-09-30

**Authors:** Harue Utsugi, Yutaka Usui, Fuyumi Nishihara, Minoru Kanazawa, Makoto Nagata

**Affiliations:** Department of Respiratory Medicine, Saitama Medical University, Morohongo 38, Moroyama-machi, Iruma-gun, Saitama Japan

**Keywords:** Hypersensitivity pneumonitis, Nontuberculous mycobacteria, Ultrasonic humidifier, Provocation test

## Abstract

**Background:**

Nontuberculous mycobacteria are well known to be a cause of hot tub lung, however, to our knowledge, there exists no case report of humidifier lung induced by mycobacteria.

**Case presentation:**

A case of a nonimmunocompromised female patient with M*ycobacterium gordonae*-induced humidifier lung is described. She spontaneously recovered after discontinuing ultrasonic humidifier use. When subjected to a provocation test, she demonstrated acute respiratory distress with signs and symptoms, consistent with hypersensitivity pneumonitis. Before and after the provocation test, water in the humidifier reservoir revealed only *Mycobacterium gordonae* by the microbiologic analyses.

**Conclusion:**

To our knowledge, this is the first report of humidifier lung induced by nontuberculous mycobacteria. Although nontuberculous mycobacteria are well-known to be agents of hot tub lung or metal working fluid lung, physicians should also consider the pathogen as a cause of hypersensitivity lung reaction associated with humidifier use.

## Background

Nontuberculous mycobacteria (NTM) are environmental microorganisms primarily residing in moist indoor environments. NTM are relatively resistant to disinfectants and grow at a wide temperature range; they are increasingly associated with pulmonary disease [1]. Among NTM, *Mycobacterium avium* is a well-known cause of hypersensitivity-like lung disease associated with hot tubs, also known as hot tub lung [2]. Other NTM are also associated with hot tub lung; *Mycobacterium fortuitum* has been implicated in a few reports of hot tub lung [3, 4], while *Mycobacterium immunogenum* may cause metal working fluid lung [5]. However, NTM-induced hypersensitivity-like lung disease or hypersensitivity pneumonitis (HP) caused by humidifier exposure has not been reported. We describe a case of *Mycobacterium gordonae*-induced humidifier lung (HL) diagnosed using a provocation test and microbiological analyses.

## Case presentation

An 89-years-old woman was evaluated for abnormal lung opacities diagnosed on thoracic computed tomography (CT) in March 2011. She reported a two-month history of exertional dyspnea with intermittent fever. She also underwent aortic valve replacement 3 years prior to the examination because of aortic valve regurgitation and had since been healthy. She was a homemaker and never-smoked. She used an ultrasonic humidifier yearly during the winter beginning 1998. Beginning in 2010, the humidifier was employed daily without cleaning the water reservoir.

On physical examination, her body temperature was 37.4 °C, and the SpO2 on room air was 84 %; otherwise normal. The white blood cell count was 10,700/μl with 73.8 % neutrophils, and the blood biochemistry results were unremarkable. The C-reactive protein was 0.28 mg/dl; KL-6, 589 U/ml; SP-D, 166 ng/ml; rheumatoid factor, 48 IU/ml; and the antibody for *Trichosporon asahii* was positive. The PaO2 on room air was 48.6 Torr. The thoracic CT showed diffuse centrilobular fine nodular infiltrates, patchy ground-glass opacities, and thickened bronchial wall predominantly in the bilateral upper lobe.

The patient was admitted for further evaluation. Pulmonary function tests revealed a forced vital capacity of 1.19 L (68.9 % predicted) and a forced expiratory volume in one second of 1.00 L (84.1 % predicted). A bronchoalveolar lavage (BAL) was performed after 7 days of hospitalization and revealed an elevated cell count of 4.2 × 10^5^/ml with 51 % lymphocytes, 3 % neutrophils, and 2 % eosinophils in the differential. The T-cell CD4^+^/CD8^+^ ratio was 0.81. Transbronchial lung biopsy revealed alveolitis with lymphocytic and plasmacytic infiltration. No pathogenic microorganisms were identified on cytology and culture of the BAL fluid. Once hospitalized, her respiratory illnesses spontaneously improved with only oxygen supplementation.

Because the symptoms arose during the winter season, humidifier lung was suspected, despite the elevated serum titer for anti-*Trichosporon asahii* antibody. The reservoir water from the ultrasonic humidifier was subjected to microbiologic analysis. Ziehl-Neelsen staining revealed abundant acid-fast bacilli on the smear, which were later identified as *M. gordonae*. In addition, *Acinetobacter iwofii, Corynebacterium* species, *Candida glabrata, Aspergillus niger,* and *Penicillium* species were cultured.

**Fig. 1:**
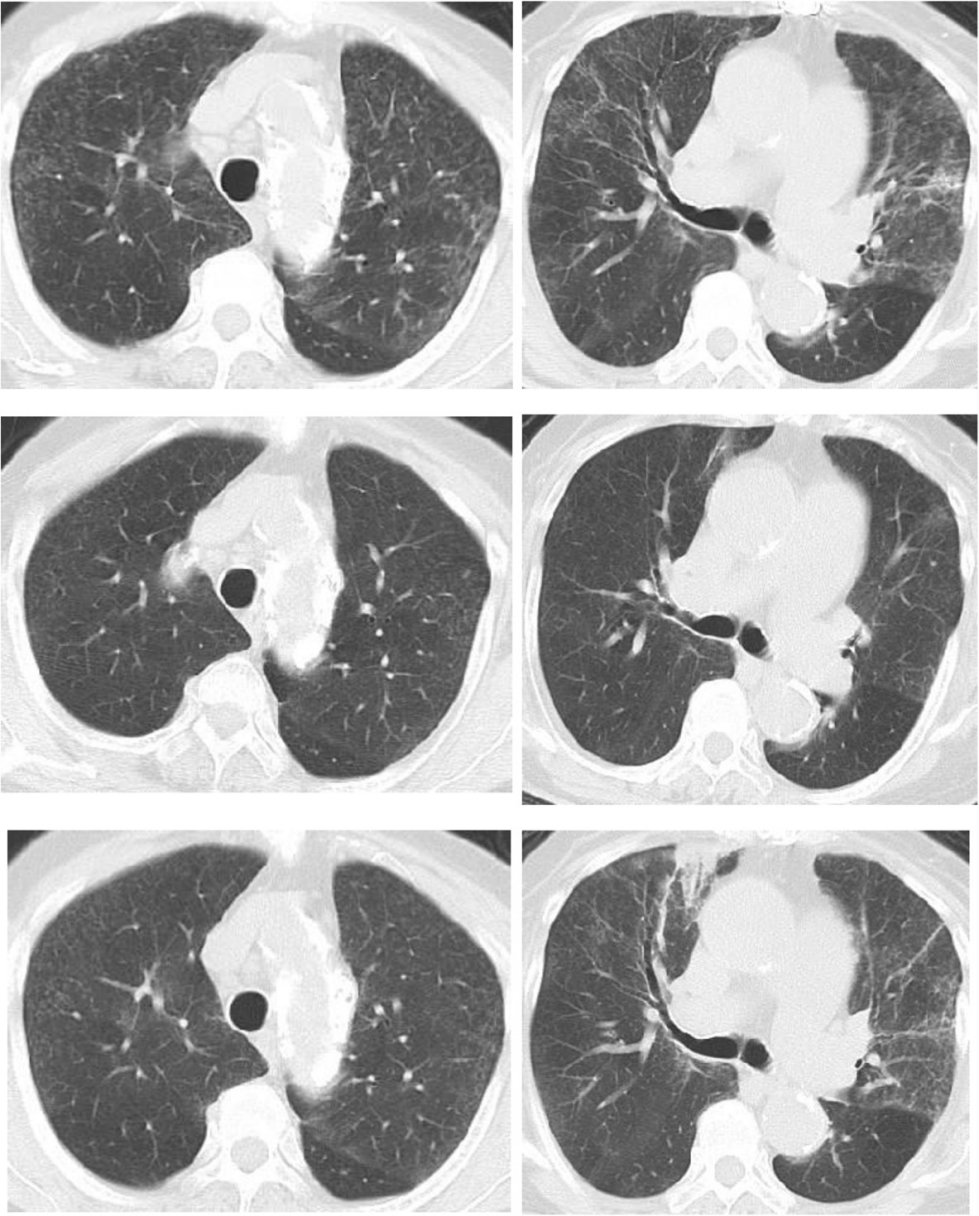
A thoracic CT before admission revealed diffuse fine centrilobular nodules with patchy ground-glass opacities of the upper lung predominance (*upper*). After the admission, the pulmonary parenchymal involvements were spontaneously improved (*middle*). 15 h after the end of the provocation test, fine centrilobular nodules and patchy ground-glass opacities appeared again. Bronchial wall thickening with peripheral airspace consolidation in the right upper lobe were also visible (*lower*)

After 15 days of hospitalization, a provocation test was performed with the humidifier. The humidifier was operated in a room measuring approximately 20 m^2^ with every window and door closed. 6 h later, the patient complained of fever and breathlessness. The provocation test was considered positive, and the patient was diagnosed with HL (Table 1 and Fig. 1). Before and immediately after the provocation test, swabs of the reservoir inner wall and the residual water were cultured, and *M. gordonae* was identified*.* No other microorganisms were detected, therefore the patient was diagnosed with *M. gordonae*-induced HL. The patient was prescribed 25 mg of oral prednisolone and rapidly recovered; she was later discharged after 30 days of hospitalization. The prednisolone dose was tapered and discontinued 2 months after discharge. She has not experienced any recurrence of symptoms since discharge. Other than avoiding use of the humidifier, no other environmental changes were required.

**Table 1:** Provocation tests by the ultrasonic humidifier

	Pre-provocation	Post-provocation
Body temperature, °C	36.8	39.4
Respiratory symptom	Dyspnea on exertion	Productive cough, Dyspnea at rest
PaO_2_/FIO_2_ ratio	302	152
FVC, L	1.28	1.00
FEV1, L	1.13	0.89
WBC count,/μl	4,760	26,560
Neutrophils, %	59.1	92.2
CRP, mg/dl	0.10	5.34

## Discussion

HL is a type of home environmental HP. In Japan, humidifiers are increasingly used to avoid excessive drying of indoor environment during winter. HL pathogens seem to depend on the humidifier type. Thermophilic actinomycetes were the most frequently isolated pathogens [6], however, recent reports of HL associated with ultrasonic humidifiers, which generate cooler vapors than evaporating- or steam- humidifiers, revealed different pathogens [7–9]. In a study evaluating ultrasonic HL, microbiological analysis of the humidifier water identified various fungi or bacteria in 4 of 5 patients (1995 Suda), however, neither thermophilic actinomycetes nor NTM were detected. In NTM-induced HP, *M. avium* is well-known cause of hot tub lung [2]. *M. fortitum* is also a reported etiology of hot tub lung in a few case reports [3, 4], and *M. immunogenum* reportedly causes metal working fluid lung [5].

In the present case, three times of microbiological analyses were conducted during admission and only *M. gordonae* was consistently identified in all the occasions. Identification of various microorganisms, including *M. gordonae*, at the first time might be related to the situation that water in the humidifier had been left as it had been for a week. After the first microbiological analysis, water in the humidifier was disposed and then restored before the provocation test. Consequently, mycobacterium, relatively resistant to drying, survived and contributed to the final diagnosis.

*M. gordonae* is a slow-growing mycobacterium and is the most frequently isolated mycobacterial contaminant. It is readily recovered from freshwater, pipelines, and laboratory faucets [1]. However, *M. gordonae* is usually nonpathogenic, though it occasionally infects immunosuppressed patients such as those with human immunodeficiency virus infection. To date, *M. gordonae*-induced hypersensitivity-like lung disease or HP has not been reported.

In this patient, the serum anti-*T. asahii* antibody was elevated. She resided in a wooden house for 20 years that was constructed 20 years ago. The low bronchoalveolar T-cell CD4^+^/CD8^+^ ratio was consistent with that for acute summer-type HP induced by *T. asahii*, which is the most common type of acute HP in Japan [10]. The positive serum antibody confirmed that she was sensitized to *T. asahii*, however, summer-type HP was unlikely for the following reasons: the condition is very rare during winter; she had no respiratory illness during summer 2010; *Trichosporon* was not detected on repetitive microbiological analyses of the humidifier reservoir water; and discontinued humidifier use prevented disease recurrence.

Some cases of *M. avium-*induced hot tub lung showed features of both lung inflammation and infection [11], however, the present case showed no evidence of infection.

## Conclusion

To the best of our knowledge, this is the first known described case of HP as well as HL induced by *M.gordonae.* Physicians should consider *M. gordonae* as a potential pathogen of ultrasonic HL in anonimmunocompromised host.

## Consent

Written informed consent was obtained from the patient for publication of this Case report and any accompanying images. A copy of the written consent is available for review by the Editor-in-Chief of this journal.

## Abbreviations

NTM: Nontuberculous mycobacteria
HP: Hypersensitivity pneumonitis
HL: Humidifier lung
CT: Computed tomography
BAL: Bronchoalveolar lavage
